# Patterns of hypnotic prescribing for residual insomnia and recurrence of major depressive disorder: a retrospective cohort study using a Japanese health insurance claims database

**DOI:** 10.1186/s12888-021-03046-z

**Published:** 2021-01-13

**Authors:** Kentaro Yamato, Ken Inada, Minori Enomoto, Tatsuro Marumoto, Masahiro Takeshima, Kazuo Mishima

**Affiliations:** 1 Japan Medical Office, Takeda Pharmaceutical Company Limited, Tokyo, Japan; 2 Department of Psychiatry, Tokyo Women’s Medical University, Tokyo, Japan; 3 Department of Medical Technology, School of Health Sciences, Tokyo University of Technology, Tokyo, Japan; 4 Department of Neuropsychiatry, Akita University Graduate School of Medicine, Akita, Japan; 5 Department of Psychophysiology, National Institute of Mental Health, National Center of Neurology and Psychiatry, Tokyo, Japan; 6 International Institute for Integrative Sleep Medicine, Tsukuba, Japan

**Keywords:** Major depressive disorder, Depression, Residual symptom, Insomnia, Recurrence, Japanese patients, Prescription pattern

## Abstract

**Background:**

Major depressive disorder (MDD) is highly prevalent in Japan and frequently accompanied by insomnia that may persist even with MDD remission. Hypnotics are used for the pharmacological treatment of insomnia, but their influence on MDD recurrence or residual insomnia following MDD remission is unclear. This retrospective, longitudinal, cohort study utilized a large Japanese health insurance claims database to investigate patterns of hypnotic prescriptions among patients with MDD, and the influence of hypnotic prescription pattern on MDD recurrence.

**Methods:**

Eligible patients (20–56 years) were those registered in the Japan Medical Data Center database between 1 January 2005 and 31 December 2018, and prescribed antidepressant and hypnotic therapy after being diagnosed with MDD. Patients who had ceased antidepressant therapy for > 180 days were followed for 1 year to evaluate depression recurrence, as assessed using Kaplan-Meier estimates. Logistic regression modelling was used to analyze the effect of hypnotic prescription pattern on MDD recurrence.

**Results:**

Of the 179,174 patients diagnosed with MDD who initiated antidepressant treatment between 1 January 2006 and 30 June 2017, complete prescription information was available for 2946 eligible patients who had been prescribed hypnotics. More patients were prescribed hypnotic monotherapy (70.8%) than combination therapy (29.2%). The most prescribed therapies were benzodiazepine monotherapy (26.2%), non-benzodiazepine monotherapy (28.9%), and combination therapy with two drugs (21.1%). Among patients prescribed multiple hypnotics, concomitant prescriptions for anxiolytics, antipsychotics, mood stabilizers and sedative antidepressants were more common. The 1-year recurrence rate for MDD was approximately 20%, irrespective of hypnotic mono- versus combination therapy or class of hypnotic therapy. Being a spouse (odds ratio [OR], 1.44; 95% confidence interval [CI], 1.03–2.02) or other family member (OR, 1.46, 95% CI, 0.99–2.16) of the insured individual, or being prescribed a sedative antidepressant (OR, 1.50, 95% CI, 1.24–1.82) conferred higher odds of MDD recurrence within 1 year of completing antidepressant therapy.

**Conclusions:**

Benzodiazepines are the most prescribed hypnotic among Japanese patients with MDD, though combination hypnotic therapy is routinely prescribed. Hypnotic prescription pattern does not appear to influence real-world MDD recurrence, though hypnotics should be appropriately prescribed given class differences in efficacy and safety.

## Background

Major depressive disorder (MDD) is highly prevalent in Japan, with a 12-month prevalence of 2.2–2.7% and lifetime prevalence of 6.1% [1, 2]. However, because only around 20% of Japanese patients with a mental disorder seek medical care [3], the prevalence of MDD may be higher than currently estimated. MDD is a leading cause of disability both globally and in Japan [4], and is associated with reduced quality of life, lower work productivity, and a substantial economic burden [5–7].

Treatment for MDD generally involves antidepressant therapy to achieve clinical remission and prevent relapse [8]. However, an estimated 20% of patients with MDD experience a recurrence within 6 months of recovery [8]. Relapse rates are particularly high among patients who discontinue treatment early [8], which may be particularly important among Japanese patients, in whom median treatment duration tends to be shorter than guideline recommendations [9].

Around one-quarter of patients with MDD have residual symptoms following remission [8], such as depressed mood, anxiety, and insomnia [10]. Importantly, residual symptoms are associated with MDD relapse and recurrence [10, 11]. Insomnia, in particular, is a common residual symptom experienced by more than 50% of patients, which, in addition to increasing the risk of MDD relapse, is associated with reduced quality of life [10, 12].

Hypnotic therapies, including benzodiazepine and non-benzodiazepine drugs, sedative–hypnotic drugs (such as sedating antidepressants), melatonin, and orexin-receptor antagonists, are used in the pharmacological treatment of insomnia [13, 14]. Hypnotic therapies are commonly co-prescribed with antidepressants to manage depression-associated insomnia [13, 15]. While hypnotic therapies can improve sleep for patients with depression-associated residual insomnia [16, 17], they have also been associated with an increased risk of developing a psychiatric disorder [18]. High doses of hypnotics have also been linked to antidepressant refractoriness and worse depression outcomes [19].

Therefore, this retrospective cohort study aimed to investigate real-world patterns of hypnotic prescribing among Japanese patients with MDD, and the influence of hypnotic prescription pattern on MDD recurrence, by interrogating the Japan Medical Data Center (JMDC) Inc. insurance claims database, which comprises accumulated health insurance receipt data (inpatient, outpatient, dispensing) from multiple health insurance associations in Japan for > 7 million individuals. The database also contains patients’ medical examination data including diagnosis and treatment information, enabling longitudinal tracking of patients across different healthcare providers.

## Methods

### Data sources, study population and cohort selection

**Fig. 1:**
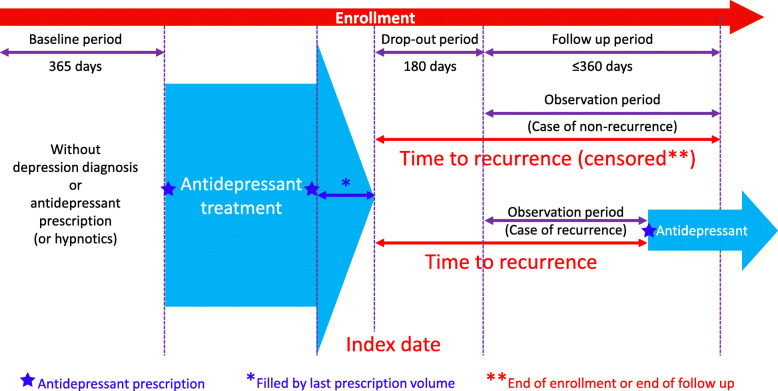
Study design

We retrospectively analyzed anonymized health insurance claims data collated in the JMDC Inc. database (system version: Netezza N2002 010 7.1.0.4.P2; Tokyo, Japan), which comprises accumulated receipts (inpatient, outpatient and dispensing) and medical examination data received from multiple health insurance associations relating to 7,175,048 people in Japan, between 1 January 2005 and 31 December 2018. Eligible patients received a diagnosis of primary or recurrent MDD (World Health Organization’s *International Statistical Classification of Diseases and Related Health Problems* [10th ed.; ICD-10] codes F32 or F33, respectively) and were prescribed antidepressant therapy, defined as a selective serotonin reuptake inhibitor (SSRI), serotonin–noradrenaline reuptake inhibitor (SNRI), noradrenergic or specific serotonergic antidepressant, or tricyclic antidepressant (World Health Organization’s *Anatomical Therapeutic Chemical* [ATC] codes N06A4, N06A5, N06A9 and N06A9, respectively) and a hypnotic (non-barbiturate, monotherapy; ATC code N05B1) between 1 January 2006 and 30 June 2017 (the study enrollment period; Fig. 1).

The index date was the date on which all antidepressant treatment was expected to be completed following dispensing of the final antidepressant prescription. Eligible patients must have had ≥1 period of ≥180 days without antidepressant treatment after the index date (the treatment completion period). Patients were also required to be aged ≥20 years at the time of initiating antidepressant therapy and < 65 years within the entire observable period from starting antidepressant therapy of up to 540 days from the index date. Patients must have had continuous health insurance enrollment for ≥12 months prior to the earliest diagnosis of depression during the study enrollment period.

Patients were excluded from the study if they had been diagnosed with MDD and not prescribed an antidepressant, or prescribed antidepressants or hypnotics without a diagnosis of MDD during the baseline period (365 days prior to first being diagnosed with MDD and prescribed an antidepressant).

### Variables

Outcomes assessed in this exploratory study were the pattern of hypnotic prescription for depression-associated insomnia, and the impact of hypnotic prescription patterns on time to MDD relapse, defined as the reissue of a prescription for antidepressant therapy after a > 180-day period without antidepressant therapy, in combination with a hypnotic for residual insomnia.

Predictor variables included patients’ demographic characteristics, insurance enrolment category (insured, spouse, or other dependent family member), prior or concomitant diagnosis of other psychiatric disorders (anxiety disorder, autism, schizophrenia, attention deficit hyperactivity disorder, bipolar disorder [ICD-10 codes: F20-F31, F40-F45, F48, F84.0, F90.0]), prior or concomitant use of other medications, (ATC codes: A01A-, A02B1, A02B2, A07E2, C01A1, C01B-, C02A1, C02A2, C02B2, C02C-, C02D-, C03A2, C03A3, C05A1, C06X-, C07A-, D05X-, D06A-, D07A-, D07B1, D07B4, D11A-, G01A1, G01A2, G03B-, G03C-, G03D-, G03E-, G03F-, G03G-, G03J-, G03X-, G04A2, H01A-, H02A1, H02A2, H02B-, H03A-, J01A-, J01C1, J01C2, J01E-, J01X9, J03A-, J04A1, J05B3, J05D1, J08B-, L01C1, L01D-, L01X9, L03B1, L03B2, L03B3, L04X-, M01A1, M02A-, M03B-, N01A2, N02B-, N03A-, N04A-, N05A9, N05BA, N05BA01-N05BA03, N05BA05, N05BA06, N05BA08, N05BA09, N05BA11, N05BA12, N05BA17-N05BA19, N05BA21-N05BA23, N05BB01, N05BE, N05B3, N07A-, N07E-, N07X-, R01A1, R02A-, R03D1, R03F1, R03L3, R05A-, R06A-, S01A-, S01B-, S01E1, S01E2, S01R-, S02C-, S03B-, S03C-), prescription for sedative antidepressant medications (mirtazapine, trazodone hydrochloride, mianserin hydrochloride), antidepressant dosage at the end of treatment (less than or greater than the recommended maximum dose), the daily imipramine-equivalent dose of antidepressant (< 75 mg/day, ≥75 and < 150 mg/day, or ≥ 150 mg/day) [20]. Prior disorders were those diagnosed < 12 months before the diagnosis and treatment of depression. Concomitant medications were those prescribed ≤6 months before the follow-up period.

Patients’ hypnotic prescription pattern was categorized as benzodiazepine monotherapy, non-benzodiazepine monotherapy, melatonin receptor agonists, orexin receptor antagonists, two agents combined, three agents combined, or ≥ 4 drugs.

### Statistical analysis

Patient demographics, clinical characteristics and study variables were summarized using descriptive statistics (mean, standard deviation [SD], median, interquartile range [IQR]). If the date of prescription was missing from the receipt, the last day of the month in which the doctor’s office visit occurred was used. If the dose of medication was missing, the data point was removed from the analysis.

The impact of hypnotic prescribing patterns on MDD recurrence during the follow-up period was assessed using Kaplan-Meier estimates.

Logistic regression modelling was used to analyze the effect of hypnotic prescription pattern on MDD relapse, with time to MDD relapse as the dependent variable, hypnotic prescription pattern as an explanatory variable, and demographic/clinical characteristics as independent variables. Adjusted odds ratios (OR) and 95% confidence intervals (CI) were calculated using a Cox proportional hazards model. A stepwise method was used to select variables to be included in the final model, using an inclusion significance level of < 0.1 and exclusion significance level of ≥0.05. A final logistic regression model was developed using age and gender (mandatory variables) and all significant predictors of MDD relapse, with the 1-year recurrence rate as the dependent variable and residual insomnia as an explanatory variable.

All statistical analyses were conducted using SAS version 9.4 (TS1M4; SAS Institute Japan Ltd., Tokyo, Japan).

## Results

**Fig. 2:**
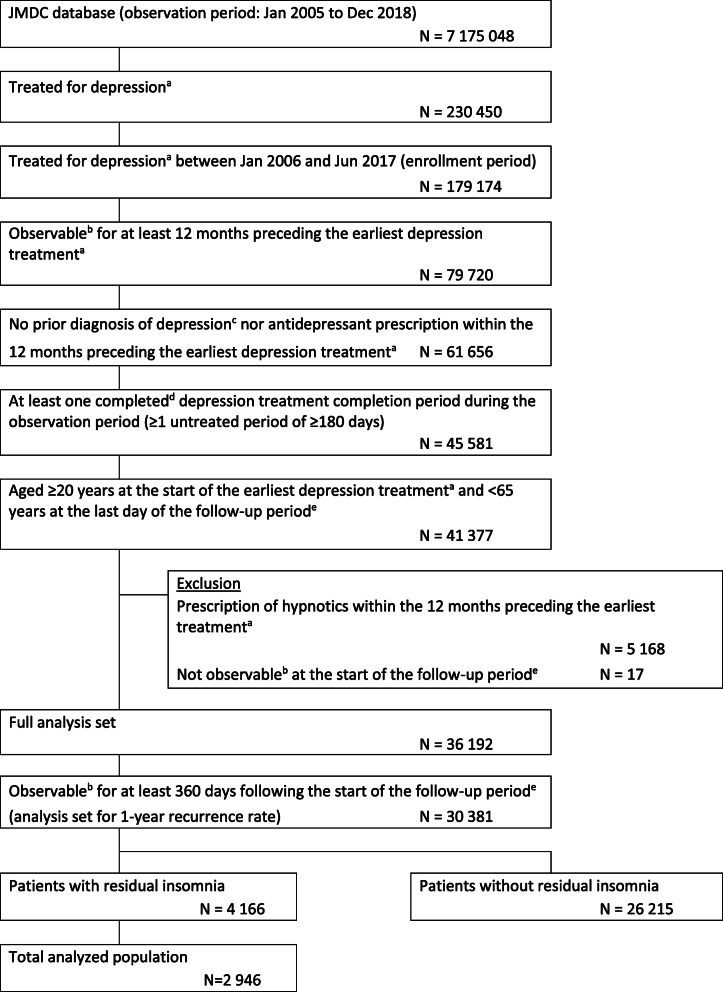
Flow diagram of study participants. ^a^A diagnosis of depression accompanied by prescription of antidepressants; ^b^Has continuous health insurance enrollment; ^c^Excluding the month immediately preceding the earliest treatment for diagnosis only; ^d^Had no antidepressant treatment for ≥180 continuous days; ^e^Up to 360 days following the last day of the first untreated period of 180 continuous days. JMDC; Japan Medical Data Center

Of the 179,174 patients diagnosed with MDD who initiated antidepressant treatment between 1 January 2006 and 30 June 2017, 36,192 patients fulfilled the eligibility criteria for this study (Fig. 2). Of those, ≥1-year follow-up data was available for 30,381 patients, of whom 4166 had been prescribed hypnotics for residual insomnia during the follow-up period. Among those prescribed hypnotics for residual insomnia, complete prescription information was available for 2946 patients (the total analyzed population).

### Patient demographics and treatment pattern

Patients in the total analyzed population (*n* = 2946) were mostly male (62.8%) and aged ≥40 years (58.8%), with a mean (SD) age of 41.6 (10.2) years (Table 1). Approximately three-quarters of patients were insured employees (74.2%). All other patients were the spouse (19.2%) or another family member (6.6%) of the insured individual. While anxiety disorder (16.6%) was the most prevalent psychiatric condition prior to MDD diagnosis, bipolar disorder (85.4%), anxiety disorder (40.7%) and schizophrenia (15.8%) were the most commonly comorbid psychiatric disorders at the time of diagnosis with MDD. Steroidal and hormonal drugs (48.8%) and anxiolytics (18.8%) were the most commonly prescribed medications prior to MDD diagnosis. At the time of diagnosis with MDD, 37.9% of patients were prescribed steroidal and hormonal drugs and concomitant anxiolytics, antipsychotics, and mood stabilizers were prescribed to 48.4, 20.6 and 7.3% of patients, respectively. Mirtazapine (20.4%) was the most frequently prescribed sedative antidepressant, followed by trazodone hydrochloride (12.2%), and mianserin hydrochloride (3.2%). At the end of treatment, almost all patients (93%) were receiving less than the maximum recommended dose of antidepressant.

**Table 1:** Patient demographics and clinical characteristics

Variable	All^**a**^ (***N*** = 2946)	Monotherapy					Combination therapy			
		Benzodiazepine (***n*** = 1067)	Non-benzodiazepine (***n*** = 850)	Melatonin receptor agonist (***n*** = 76)	Orexin receptor antagonist (***n*** = 94)	Total (***n*** = 2087)	2 drugs (***n*** = 622)	3 drugs (***n*** = 166)	≥4 drugs (***n*** = 71)	Total (***n*** = 859)
**Age, years, mean ± SD**	41.6 ± 10.2	42.4 ± 9.9	42.2 ± 10.0	38.3 ± 11.2	42.5 ± 11.4	42.2 ± 10.1	40.5 ± 10.4	38.9 ± 10.1	38.9 ± 9.1	40.1 ± 10.2
< 40 years, n (%)	1214 (41.2)	404 (37.9)	328 (38.6)	37 (48.7)	40 (42.6)	809 (38.8)	284 (45.7)	84 (50.6)	37 (52.1)	405 (47.1)
≥ 40 years, n (%)	1732 (58.8)	663 (62.1)	522 (61.4)	39 (51.3)	54 (57.4)	1278 (61.2)	338 (54.3)	82 (49.4)	34 (47.9)	454 (52.9)
**Sex, n (%)**										
Male	1850 (62.8)	693 (64.9)	507 (59.6)	44 (57.9)	51 (54.3)	1295 (62.1)	403 (64.8)	105 (63.3)	47 (66.2)	555 (64.6)
**Insured or dependent, n (%)**										
Insured	2186 (74.2)	805 (75.4)	619 (72.8)	49 (64.5)	68 (72.3)	1541 (73.8)	468 (75.2)	124 (74.7)	53 (74.6)	645 (75.1)
Spouse	567 (19.2)	201 (18.8)	187 (22.0)	14 (18.4)	25 (26.6)	427 (20.5)	103 (16.6)	24 (14.5)	13 (18.3)	140 (16.3)
Other family member	193 (6.6)	61 (5.7)	44 (5.2)	13 (17.1)	1 (1.1)	119 (5.7)	51 (8.2)	18 (10.8)	5 (7.0)	74 (8.6)
**History of psychiatric disorders**^**b**^**, n (%)**										
Anxiety disorder	489 (16.6)	179 (16.8)	155 (18.2)	14 (18.4)	16 (17.0)	364 (17.4)	89 (14.3)	23 (13.9)	13 (18.3)	125 (14.6)
Bipolar disorder	14 (0.5)	3 (0.3)	8 (0.9)	1 (1.3)	0	12 (0.6)	2 (0.3)	0 (0)	0	2 (0.2)
Schizophrenia	40 (1.4)	18 (1.7)	5 (0.6)	1 (1.3)	0	24 (1.1)	10 (1.6)	6 (3.6)	0	16 (1.9)
ADHD	2 (0.1)	0	0	0	0	0	2 (0.3)	0	0	2 (0.2)
Autism	1 (0.0)	0	0	0	0	0	1 (0.2)	0	0	1 (0.1)
**Comorbid psychiatric disorders**^**c**^**, n (%)**										
Anxiety disorder	1200 (40.7)	416 (39.0)	343 (40.4)	36 (47.7)	45 (47.9)	840 (40.2)	263 (42.3)	62 (37.3)	35 (49.3)	360 (41.9)
Bipolar disorder	509 (17.3)	167 (15.7)	78 (9.2)	10 (13.2)	13 (13.8)	268 (12.8)	166 (26.7)	47 (28.3)	28 (39.4)	241 (28.1)
Schizophrenia	466 (15.8)	179 (16.8)	61 (7.2)	6 (7.9)	13 (13.8)	259 (12.4)	138 (22.2)	47 (28.3)	22 (31.0)	207 (24.1)
ADHD	48 (1.6)	16 (1.5)	4 (0.5)	2 (2.6)	3 (3.2)	25 (1.2)	13 (2.1)	9 (5.4)	1 (1.4)	23 (2.7)
Autism	2 (0.1)	0	0	0	0	0	2 (0.3)	0	0	2 (0.2)
**History of medication at baseline**^**b**^**, n (%)**										
Steroid, hormonal drug	1437 (48.8)	501 (47.0)	452 (53.2)	36 (47.4)	53 (56.4)	1042 (49.9)	293 (47.1)	73 (44.0)	29 (40.8)	395 (46.0)
Anxiolytics	555 (18.8)	201 (18.8)	176 (20.7)	15 (19.7)	18 (19.1)	410 (19.6)	101 (16.2)	31 (18.7)	13 (18.3)	145 (16.9)
Leukotriene receptor antagonists	276 (9.4)	96 (9.0)	82 (9.6)	6 (7.9)	9 (9.6)	193 (9.2)	54 (8.7)	21 (12.7)	8 (11.3)	83 (9.7)
Neurological medication, psychotropic drug	198 (6.7)	66 (6.2)	60 (7.1)	2 (2.6)	5 (5.3)	133 (6.4)	43 (6.9)	15 (9.0)	7 (9.9)	65 (7.6)
Analgesic, anti-inflammatory	187 (6.3)	67 (6.3)	55 (6.5)	1 (1.3)	8 (8.5)	131 (6.3)	44 (7.1)	11 (6.6)	1 (1.4)	56 (6.5)
Antibacterial and/or antifungal	179 (6.1)	56 (5.2)	53 (6.2)	5 (6.6)	4 (4.3)	118 (5.7)	41 (6.6)	15 (9.0)	5 (7.0)	61 (7.1)
Antihypertensive, cardiotonic	52 (1.8)	14 (1.3)	22 (2.6)	3 (3.9)	1 (1.1)	40 (1.9)	8 (1.3)	4 (2.4)	0	12 (1.4)
Antipsychotic	49 (1.7)	18 (1.7)	13 (1.5)	1 (1.3)	0	32 (1.5)	10 (1.6)	7 (4.2)	0	17 (2.0)
Antismoking product	21 (0.7)	9 (0.8)	5 (0.6)	0	0	14 (0.7)	7 (1.1)	0	0	7 (0.8)
Antineoplastic	7 (0.2)	2 (0.2)	3 (0.4)	1 (1.3)	1 (1.1)	7 (0.3)	0	0	0	0
Mood stabilizer	2 (0.1)	1 (0.1)	1 (0.1)	0	0	2 (0.1)	0	0	0	0
Immunostimulant	2 (0.1)	2 (0.2)	0	0	0	2 (0.1)	0	0	0	0
Centrally-acting anti-obesity product	1 (0)	0	0	0	0	0	1 (0.2)	0	0	1 (0.1)
Other	324 (11.0)	124 (11.6)	92 (10.8)	9 (11.8)	14 (14.9)	239 (11.5)	56 (9.0)	19 (11.4)	10 (14.1)	85 (9.9)
**Concomitant medication at baseline**^**c**^**, n (%)**										
Steroid, hormonal drug	1118 (37.9)	383 (35.9)	353 (41.5)	27 (35.5)	40 (42.6)	803 (38.5)	229 (36.8)	55 (33.1)	31 (43.7)	315 (36.7)
Anxiolytics	1427 (48.4)	521 (48.8)	361 (42.5)	39 (51.3)	47 (50.0)	968 (46.4)	319 (51.3)	96 (57.8)	44 (62.0)	459 (53.4)
Leukotriene receptor antagonists	173 (5.9)	60 (5.6)	58 (6.8)	5 (6.6)	6 (6.4)	129 (6.2)	27 (4.3)	12 (7.2)	5 (7.0)	44 (5.1)
Neurological medication, psychotropic drug	260 (8.8)	92 (8.6)	54 (6.4)	7 (9.2)	6 (6.4)	159 (7.6)	60 (9.6)	24 (14.5)	17 (23.9)	101 (11.8)
Analgesic, anti-inflammatory	96 (3.3)	36 (3.4)	26 (3.1)	2 (2.6)	1 (1.1)	65 (3.1)	22 (3.5)	9 (5.4)	0	31 (3.6)
Antibacterial and/or antifungal	115 (3.9)	34 (3.2)	33 (3.9)	2 (2.6)	6 (6.4)	75 (3.6)	27 (4.3)	9 (5.4)	4 (5.6)	40 (4.7)
Antihypertensive, cardiotonic	82 (2.8)	26 (2.4)	26 (3.1)	2 (2.6)	2 (2.1)	56 (2.7)	17 (2.7)	7 (4.2)	2 (2.8)	26 (3.0)
Antipsychotic	607 (20.6)	222 (20.8)	89 (10.5)	10 (13.2)	16 (17.0)	337 (16.1)	178 (28.6)	59 (35.5)	33 (46.5)	270 (31.4)
Antismoking product	17 (0.6)	3 (0.6)	4 (0.5)	0	1 (1.1)	8 (0.4)	4 (0.6)	4 (2.4)	1 (1.4)	9 (1.0)
Antineoplastic	12 (0.4)	4 (0.4)	4 (0.5)	1 (1.3)	1 (1.1)	10 (0.5)	1 (0.2)	0	1 (1.4)	2 (0.2)
Mood stabilizer	215 (7.3)	63 (5.9)	27 (3.2)	5 (6.6)	4 (4.3)	99 (4.7)	73 (11.7)	26 (15.7)	17 (23.9)	116 (13.5)
Immunostimulant	1 (0)	1 (0.1)	0	0	0	1 (0.0)	0	0	0	0
Centrally-acting anti-obesity product	2 (0.1)	0	0	0	0	0	1 (0.2)	1 (0.6)	0	2 (0.2)
Other	231 (7.8)	80 (7.5)	68 (8.0)	4 (5.3)	7 (7.4)	159 (7.6)	48 (7.7)	19 (11.4)	5 (7.0)	72 (8.4)
**Sedative antidepressant, n (%)**	917 (31.1)	288 (27.0)	251 (29.5)	26 (34.2)	38 (40.4)	603 (28.9)	221 (35.5)	65 (39.2)	28 (39.4)	314 (36.6)
Mirtazapine	600 (20.4)	177 (16.6)	165 (19.4)	20 (26.3)	26 (27.7)	388 (18.6)	149 (24.0)	43 (25.9)	20 (28.2)	212 (24.7)
Trazodone hydrochloride	360 (12.2)	122 (11.4)	87 (10.2)	10 (13.2)	12 (12.8)	231 (11.1)	88 (14.1)	27 (16.3)	14 (19.7)	129 (15.0)
Mianserin hydrochloride	95 (3.2)	31 (2.9)	19 (2.2)	1 (1.3)	4 (4.3)	55 (2.6)	28 (4.5)	8 (4.8)	4 (5.6)	40 (4.7)
**Antidepressant dosage at end of treatment, n (%)**										
< Recommended maximum dose	2740 (93.0)	987 (92.5)	806 (94.8)	70 (92.1)	91 (96.8)	1954 (93.6)	570 (91.6)	150 (90.4)	66 (93.0)	786 (91.5)
≥ Recommended maximum dose	206 (7.0)	80 (7.5)	44 (5.2)	6 (7.9)	3 (3.2)	133 (6.4)	52 (8.4)	16 (9.6)	5 (7.0)	73 (8.5)
**Dosage of antidepressants during treatment (mg/day)**^**d**^**, n (%)**										
Mean (95% CI)	74.3 (72.3–76.3)	73.0 (69.9–76.1)	68.2 (64.8–71.7)	67.3 (52.9–81.7)	69.1 (57.9–80.4)	70.7 (68.4–72.9)	82.5 (77.4–87.7)	87.7 (77.7–97.7)	77.4 (66.6–88.3)	83.1 (78.8–87.4)
Median (IQR)	64.9 (37.5, 100.0)	65.5 (37.5, 100.0)	58.4 (37.5, 81.2)	46.4 (37.5, 75.0)	60.2 (30.0. 85.7)	61.1 (37.5, 93.5)	71.0 (37.5, 105.1)	75.0 (37.5, 120.1)	75.0 (38.1, 105.0)	73.0 (37.5, 106.8)
< 75 mg/day	1678 (57.0)	605 (56.7)	526 (61.9)	53 (69.7)	54 (57.4)	1238 (59.3)	328 (52.7)	80 (48.2)	32 (45.1)	440 (51.2)
≥ 75 and < 150 mg/day	1005 (34.1)	378 (35.4)	264 (31.1)	16 (21.1)	32 (34.0)	690 (33.1)	218 (35.0)	62 (37.3)	35 (49.3)	315 (36.7)
≥ 150 mg/day	263 (8.9)	84 (7.9)	60 (7.1)	7 (9.2)	8 (8.5)	159 (7.6)	76 (12.2)	24 (14.5)	4 (5.6)	104 (12.1)

^a^Analysis set for 1-year recurrence rate; ^b^Within 12 months prior to treatment for depression; ^c^Within 6 months before start of the follow-up period; ^d^Converted to values that are equivalent to imipramine using a dose-equivalence scale for antidepressants [20].

*ADHD* Attention deficit hyperactivity disorder, *CI* Confidence interval, *IQR* Interquartile range, *SD* Standard deviation

The vast majority of patients (70.8%) were prescribed hypnotic monotherapy rather than combination therapy comprising ≥2 drugs (29.2%). Most prescriptions were for benzodiazepine monotherapy (36.2%) and non-benzodiazepine monotherapy (28.9%), followed by combination therapy with two drugs (21.1%). Patient demographics and clinical characteristics among hypnotic prescription subgroups were generally comparable to the total analyzed population regarding age, male sex, insurance type, prior and concomitant psychiatric disorders and medications, sedative antidepressant prescriptions, and antidepressant dosage at the end of treatment. Similarly, patient demographics and clinical characteristics were generally comparable between patients prescribed monotherapy versus combination therapy, except that a greater proportion of patients prescribed hypnotic monotherapy were aged ≥40 years (61.2% vs 52.9%), and more patients prescribed combination hypnotic therapy had comorbid schizophrenia (24.1% vs 12.4%). Furthermore, concomitant prescriptions for antipsychotics and mood stabilizers were more common among patients prescribed combination hypnotic therapy (31.4 and 13.5%) versus monotherapy (16.1 and 4.7%). Specifically, the rate of concomitant antipsychotic prescriptions was 28.6, 35.5 and 46.5% among patients prescribed two-, three- or ≥ 4-drug combination hypnotic therapy, respectively, versus 20.6% overall. Similarly, while 7.3% of patients were prescribed mood stabilizers overall, combination hypnotic therapies were associated with mood stabilizer prescription rates of 11.7, 15.7 and 23.9%, respectively. Concomitant prescriptions for anxiolytics also tended to be higher among patients prescribed three-drug (57.8%) or ≥ 4-drug (62.0%) combination hypnotic therapy. Prescriptions for sedative antidepressants also tended to be higher among patients prescribed two- (24.0%), three- (25.9%) or ≤ 4-drug (28.2%) combination hypnotic therapies; a similar pattern was observed with trazodone hydrochloride and mianserin hydrochloride prescriptions.

Overall, mean antidepressant dosage was notably higher among patients prescribed combination therapy than monotherapy (83.1 vs 70.7 mg/day). Specifically, mean antidepressant dosage was higher among patients prescribed two- (82.5 imipramine-equivalent mg/day), three- (87.7 imipramine-equivalent mg/day), or ≥ 4-drug (77.4 imipramine-equivalent mg/day) combination hypnotic therapy compared with the total analyzed population (74.3 imipramine-equivalent mg/day).

### Effect of hypnotic prescription pattern on MDD recurrence

**Fig. 3:**
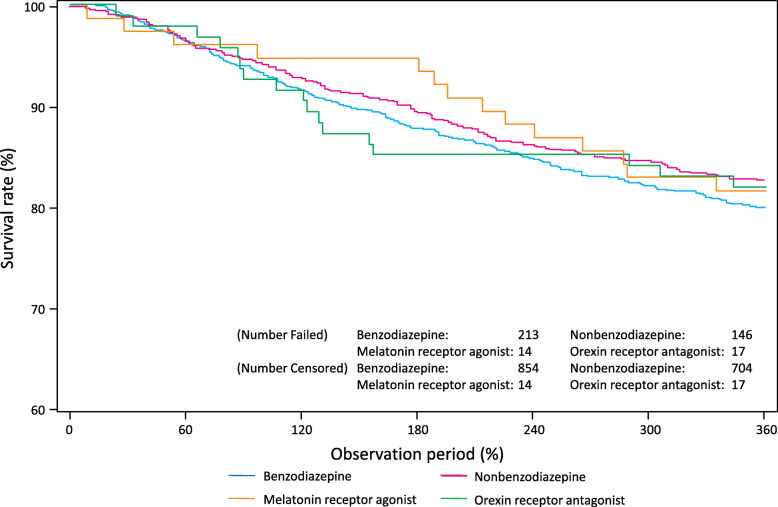
Time to MDD recurrence in patients prescribed hypnotic monotherapy for residual insomnia. MDD, major depressive disorder

There were no differences in MDD recurrence rates between patients prescribed monotherapy versus combination therapy for residual insomnia, or according to class of hypnotic prescribed (Fig. 3). Specifically, 1-year recurrence rates were 20% (*n* = 213) with benzodiazepine, 17.2% (*n* = 146) with non-benzodiazepine, 18.4% (n = 14) with melatonin receptor agonist and 18.1% (*n* = 17) with orexin receptor antagonist therapy. Recurrence rates were 18.7% (*n* = 390) with hypnotic monotherapy versus 23.3% (*n* = 200) with combination treatment. Given the intersecting natures of the Kaplan-Meier curves (Fig. 3), we did not conduct log-rank and Wilcoxon tests to compare recurrence rates between groups.

### Factors associated with MDD recurrence

Among patients prescribed hypnotic therapy for depression-associated residual insomnia, the odds of MDD recurrence within 1 year of completing antidepressant therapy were higher among those who were a spouse (OR, 1.44; 95% CI, 1.03–2.02) or other family member (OR, 1.46; 95% CI, 0.99–2.16) of the insured individual, and those who were prescribed a sedative antidepressant (OR, 1.50, 95% CI, 1.24–1.82; Table 2), but lower among females (OR, 0.67; 95% CI, 0.50–0.88). In contrast, prescription pattern did not influence the odds of MDD recurrence.

**Table 2:** Multivariate analysis for predictors of MDD recurrence

Variable	Reference	Category	MDD recurrence at 360 days, OR (95% CI)
Prescription pattern	Benzodiazepine	Non-benzodiazepine	0.84 (0.67–1.07)
		Melatonin receptor agonist	0.87 (0.47–1.60)
		Orexin receptor antagonist	0.87 (0.50–1.50)
		Combination therapy (2 drugs)	1.23 (0.97–1.56)
		Combination therapy (3 drugs)	0.91 (0.60–1.37)
		Combination therapy (≥4 drugs)	0.97 (0.53–1.75)
Gender^a^	Male	Female	0.67 (0.50–0.88)
Age^a^	< 40 years	≥40 years	0.95 (0.78–1.14)
Insured or dependent	Insured	Spouse	1.44 (1.03–2.02)
		Other family member	1.46 (0.99–2.16)
Prior neurological medication, psychotropic drug	Absent	Present	1.22 (0.86–1.73)
Prior anxiolytic medication	Absent	Present	0.98 (0.77–1.24)
Concomitant antipsychotic medication	Absent	Present	1.11 (0.88–1.39)
Sedative antidepressant	Absent	Present	1.50 (1.24–1.82)
Antidepressant dosage at end of treatment	<Recommended maximum dose	≥Recommended maximum dose	1.11 (0.77–1.60)
Antidepressant dosage (mg/day)^b^	< 75 mg/day	≥75 and < 150 mg/day	1.06 (0.86–1.29)
		≥150 mg/day	1.14 (0.81–1.61)

^a^Mandatory variables; ^b^Converted to values that are equivalent to imipramine using a dose-equivalence scale for antidepressants [20].

*CI* Confidence interval, *MDD* Major depressive disorder, *OR* Odds ratio

## Discussion

This retrospective study utilized a large health insurance claims database to explore hypnotic prescription patterns and depression recurrence among Japanese patients with MDD. The findings illustrate that among patients with MDD prescribed hypnotic therapy, most were prescribed monotherapy after ceasing antidepressant therapy, predominantly benzodiazepines, while about one-third of patients were prescribed combination hypnotic therapy comprising ≥2 drugs. Importantly, concomitant prescriptions for anxiolytics, antipsychotics, mood stabilizers and sedative antidepressants were more common among patients prescribed multiple hypnotic therapies.

In patients prescribed combination hypnotic therapy, antidepressant dosages were substantially higher compared with patients prescribed monotherapy, which may reflect greater severity of depression or antidepressant-resistant depression in patients prescribed combination therapy. While severe depression is considered a risk factor for MDD recurrence [8, 21], there was no difference in recurrence rates according to hypnotic prescription in this study. Accordingly, hypnotic combination therapy may have prevented MDD recurrence in patients with severe depression.

While a previous study reported a significantly higher risk of depressive disorders over 6 years with long-term sedative–hypnotic prescriptions, including benzodiazepines in patients with insomnia [18], the current findings suggest depression recurrence among Japanese patients is unaffected by hypnotic prescription over a shorter, 1-year period. Likewise, no differences in MDD recurrence rates was observed between patients prescribed hypnotic monotherapy versus combination therapy, or between classes of hypnotic therapy. However, it must be noted that sedatives and hypnotics are considered to be a short-term solution for managing insomnia, and this study has investigated their use in this context.

These findings confirm the relatively high rates of psychotropic polypharmacy in Japan [9, 22, 23]. Although Japanese treatment guidelines for MDD recommend antidepressant monotherapy, in reality around three-quarters of patients with MDD are prescribed multiple therapies [9]. Benzodiazepines formed the backbone of antidepressant therapy and combination therapy in this study, in concordance with benzodiazepine monotherapy being the preferred initial treatment for MDD among Japanese clinicians [24], and a combination of first- and/or second-generation antidepressants and benzodiazepines were the predominant polypharmacy [9].

That hypnotic therapy with two drugs was more common than ≥3-drug regimens is perhaps not surprising, and may reflect the reduced payments from public insurance payers to medical institutions if ≥3 hypnotics are simultaneously prescribed [22]. Likewise, our observation that almost all patients were prescribed less than the recommended maximum dose of antidepressants is in keeping with prescribing practices in Japan [22].

Therefore, if depression-associated insomnia can be effectively managed using fewer medications, without increasing the risk of depression recurrence, then combination therapy – with its potential for drug–drug interactions – should be avoided whenever possible. Furthermore, consideration should be given to the hypnotic prescribed for depression-associated insomnia because efficacy and safety varies across classes. For example, benzodiazepines are associated with multiple adverse events, including cognitive and psychomotor impairment, amnesia, next-day hangover, rebound insomnia and dependence [13]. In contrast, newer hypnotic therapies including melatonin agonists, improve sleep without producing residual sedation, or memory, cognitive or psychomotor impairment [13]. In this study, no difference in MDD recurrence was found by hypnotic drug type, suggesting that these advantages may be realized without increasing risk of depression relapse in patients with residual insomnia. However, a recent meta-analysis has reported differences in MDD relapse rates according to class of antidepressant therapy [25].

This study is limited by the JMDC database only providing data for employees aged 20–64 years and their spouse or family members, and the majority of study subjects (62.8%) were male, meaning the results may not be generalizable to the overall Japanese population. Furthermore, the database does not capture the criteria used to diagnose patients with MDD and the severity of depression, which could influence diagnostic and prescribing patterns. Likewise, patients with severe depression have a higher risk of being unemployed, and would therefore not be captured in the JMDC database [26], which could be evidenced by the higher odds of recurrence in spouses and other family members versus insured employees observed in this study.

Similarly, a filled prescription claim does not indicate consumption of, or adherence to, prescription medication and it was assumed that patients who discontinued antidepressant treatment were in remission, as opposed to failing to return to a healthcare provider to refill their prescription. Likewise, it was inferred that patients were prescribed hypnotic therapy during follow-up to manage depression-associated residual insomnia.

## Conclusions

The rates of MDD recurrence in patients receiving hypnotics for residual insomnia was not affected by hypnotic monotherapy versus combination therapy, or class of hypnotic therapy, indicating that choice of ongoing treatment for insomnia is unlikely to increase the risk of recurrent MDD once antidepressant therapy ceases for real-world Japanese patients. Benzodiazepine monotherapy is the most commonly prescribed hypnotic among Japanese patients with MDD, though a substantial proportion of patients are prescribed ≥2 hypnotic therapies. Hypnotics should be appropriately prescribed for depression-associated insomnia, given differences in efficacy and safety across classes of hypnotics. Newer classes of hypnotics may be useful for avoiding some of the drawbacks of benzodiazepine therapy.

## Data Availability

The data set used for analysis in this study is available from JMDC Inc. but were used under license for the current study. Restrictions thus apply, and the data are not publicly available. For inquiries about access to the data set used in this study, please contact JMDC (https://www.jmdc.co.jp).

## Abbreviations

ATC: Anatomical Therapeutic Chemical
CI: Confidence interval
ICD-10: International Statistical Classification of Diseases and Related Health Problems (10th ed.)
IQR: Interquartile range
JMDC: Japan Medical Data Center
MDD: Major depressive disorder
OR: Odds ratio
SD: Standard deviation
SNRI: Serotonin–noradrenaline reuptake inhibitor
SSRI: Selective serotonin reuptake inhibitor
